# Intelligent Task Dispatching and Scheduling Using a Deep Q-Network in a Cluster Edge Computing System

**DOI:** 10.3390/s22114098

**Published:** 2022-05-28

**Authors:** Joosang Youn, Youn-Hee Han

**Affiliations:** 1 Department of Industrial ICT Engineering, Dong-Eui University, Busan 47340, Korea; 2 Future Convergence Engineering, Department of Computer Science and Engineering, Korea University of Technology and Education, Cheonan 31253, Korea; yhhan@koreatech.ac.kr

**Keywords:** edge computing, task offloading, deep reinforcement learning, clustering

## Abstract

Recently, intelligent IoT applications based on artificial intelligence (AI) have been deployed with mobile edge computing (MEC). Intelligent IoT applications demand more computing resources and lower service latencies for AI tasks in dynamic MEC environments. Thus, in this paper, considering the resource scalability and resource optimization of edge computing, an intelligent task dispatching model using a deep Q-network, which can efficiently use the computing resource of edge nodes is proposed to maximize the computation ability of the cluster edge system, which consists of multiple edge nodes. The cluster edge system can be implemented with the Kubernetes technology. The objective of the proposed model is to minimize the average response time of tasks offloaded to the edge computing system and optimize the resource allocation for computing the offloaded tasks. For this, we first formulate the optimization problem of resource allocation as a Markov decision process (MDP) and adopt a deep reinforcement learning technology to solve this problem. Thus, the proposed intelligent task dispatching model is designed based on a deep Q-network (DQN) algorithm to update the task dispatching policy. The simulation results show that the proposed model archives a better convergence performanc in terms of the average completion time of all offloaded tasks, than existing task dispatching methods, such as the Random Method, Least Load Method and Round-Robin Method, and has a better task completion rate than the existing task dispatching method when using the same resources as the cluster edge system.

## 1. Introduction

Recently, massive computing resource-consuming and delay-sensitive-based diverse IoT applications have been emerging. These applications are deployed with a computation offloading scheme, which offloads the tasks to an edge with enough computing resources. In addition, these applications demand more computing resources. Thus, some solutions are emerging that can flexibly use computing resources in edge computing [1,2,3]. Solutions based on mobile edge computing (MEC) [4,5] or fog computing [6] deploy IoT services with computation offloading at the edge networks. These solutions can obtain a better quality of service (QoS), such as a fast task response time for computation-intensive and latency-sensitive IoT applications such as augmented reality, virtual reality, and object detection [7,8]. To strictly satisfy the quality of service requested by IoT applications, a feasible solution is proposed to offload part of the tasks to the remote cloud for collaborative processing and return the task results to the edge computing server. Although the cloud provides enough computing resources, the offloading tasks sent to the cloud would suffer unpredictable delays due to network congestion, fail to meet the task response time deadline required by a task and degrade the quality of service. Thus, to overcome these shortcomings, solutions using collaborative resource allocation in a distributed computing manner between the edge computing server and the cloud have been proposed [9,10,11,12,13]. Using the task scheduling method for collaborative resource allocation between the edge computing server and the cloud is more challenging for several reasons. First, the link transmission delay is stochastic due to the dynamic network conditions between the edge server and the cloud. Second, response times are different depending on the available resources. Lastly, the task arrival rate, task size, and task return time requirement are different for diverse IoT applications, making task-scheduling to ensure the optimal collaborative manner between the edge computing server and the cloud more challenging [12].

In addition, it is necessary to consider the resource scalability of edge computing. Recently, more research has adopted Kubernetes technology for the resource scalability of edge computing [14,15,16]. Kubernetes is an open-source platform which is optimized to configure the infrastructures to deploy the cluster-based edge system due to its inherent portability and scalability [14]. It also enables the automated deployment, scaling, and management of containerized applications. It is based on a master–worker architecture, where a kubelet is used as the communication interface between the master and workers [15]. When deploying a private cloud and cluster-based edge system in several industry domains, the Kubernetes technique is used to increase the resource scalability [16,17].

In this paper, we use a network model based on the cluster edge system implemented with the clustering technique [14]. Compared with the existing non-cluster edge system with resource scalability limitations, the cluster edge system implemented based on the Kubernetes technology has the advantage of being able to flexibly use the computing resources of the cluster edge system by adding the number of edge nodes within a cluster. However, at present, the resource allocation policy used for the offloaded tasks at the cluster edge system has used traditional resource allocation policies such as the random-based policy, least-load-based policy, and the round-robin-based policy. These traditional resource allocation polices cannot ensure quality of service, such as the task response time demanded from IoT applications in dynamic MEC environments. Thus, in our work, we focus on the optimization problem of resource allocation to maximize computation capabilities in the cluster edge system and propose an optimal resource allocation policy considering the resource scalability and resource optimization in a cluster edge system. Specifically, we aim to address how to estimate the load status of edge nodes in the cluster edge in the dynamic environment and find the edge node that can ensure the demanded quality of service without overload and congestion for task dispatching to minimize the average response time of computation tasks offloaded to the edge computing system and allocate optimal resources to compute offloaded tasks. To achieve these objectives, we propose an intelligence task dispatching method using the deep Q-network, which can efficiently use the computing resources in the cluster edge system. The goal of the proposed method is to minimize the average task response time through minimizing the completion time of tasks offloaded to the cluster edge system and achieve a high task completion rate by allocating the optimal resources. We first formulated the optimization problem of resource allocation as a Markov decision process (MDP), which is an effective approach to model the sequential decision-making problem, and then, for the MDP-based optimization problem, a deep Q-network (DQN) algorithm is proposed as the reinforcement learning (RL) learning technology to find the optimal policy in the proposed edge network.

In this paper, we investigate the collaborative computing resource allocation problem with the objective of minimizing the average task service delay and maintaining fairness and efficiency in terms of the utility of computing resources in the cluster edge. The main contributions of this paper are summarized as follows:

1 The cluster edge system is investigated using the clustering technique. Compared with the existing non-cluster edge system with the limitations of resource scalability, the cluster edge system has the advantage of being able to flexibly use the computing resources of the cluster edge system by joining the number of edge nodes within a cluster.
2 The optimization problem related to the resource allocation policy for the cluster edge system is formulated. The aim is to optimize the resource allocation policy considering the resource scalability and resource optimization in a cluster edge system. The formulated problem is based on the Markov decision process (MDP), which is solved by our proposed deep Q-network (DQN) optimization algorithm.
3 A deep Q-network (DQN)-based intelligent task dispatching method is proposed. To evaluate the proposed model, a mathematical model-based simulator is developed. The simulations for a performance evaluation are to validate the mathematical formulation and the DQN based algorithm for task dispatching. Simulation results show that the proposed method can achieve the optimal performance for average task service delays and average task completion rate in terms of the utility of computing resources in the cluster edge system.

The rest of the paper is organized as follows. Section 2 presents the related works and Section 3 provides the system model and problem statement. Section 4 proposes a solution for the problem and an intelligent task dispatching method using a deep Q-network (DQN) algorithm. In Section 5, the performance evaluation of the proposed method is presented. Finally, this paper is concluded in Section 6.

## 2. Related Work

Recently, there have been several studies regarding resource allocation schemes that adopted a deep reinforcement learning algorithm in an edge computing environment [9,10,11,12,13,18]. In [9], assuming that there are mobile edge computing networks (MECNs) consisting of multiple access points; multi-edge servers; and N mobile nodes, where each mobile node has M independent, real-time massive tasks; a reinforcement-learning-based state-action-reward-state-action (RL-SARSA) algorithm is proposed to resolve the resource management problem and make the optimal offloading decision to minimize system costs, including energy consumption and computing time delays. In [10], a deep reinforcement learning algorithm is proposed to solve the collaborative computation offloading problem in the heterogeneous edge computing environment. In [11], an improved deep Q-network (DQN)-algorithm-based resource allocation policy is proposed for the IoT edge computing system to improve the efficiency of resource utilization and minimize the task completion delay. The proposed method formulates the resource allocation problem as the MDP and proposes an improved DQN algorithm to learn the resource allocation policy, which can use multiple replay memories. In [12], an intelligent resource allocation framework (iRAF) is proposed to support massive resource-consuming and delay-sensitive IoT services in edge computing. The Monte Carlo tree search (MCTS)-algorithm-based iRAF automatically learns the dynamic network environment and generates resource allocation decisions to maximize the performance over service delay and power consumption in the collaborative mobile edge computing network. In [13], an intelligent task scheduling framework focusing on heterogeneous VM resource allocation is proposed in IoT edge computing environments, where to solve the task scheduling problem, a policy-based reinforce algorithm is adopted. In [18], a distributed task migration algorithm based on counterfactual multi-agent reinforcement learning is proposed for task migration optimization in MEC. Specifically, the proposed algorithm in [18] can facilitate cooperation among users with low computational complexity. In [19], the IoT devices’ offloading decision method is proposed to solve CPU frequencies and transmit powers’ joint optimization problem for an MEC environment. The proposed method uses a mixed integer nonlinear program (MINLP) algorithm to minimize the sum of the computing pressure on the primary MEC server, the sum of the energy consumption of the network, and the task dropping cost. In addition, the DRL-based optimization algorithm is developed to solve the nonconvex problem. In [20], a DRL-based dynamic resource management scheme is proposed to minimize the average service delay of the offloaded tasks in an industrial IoT MEC network. In [21], a DRL-based resource allocation method for computation offloading is proposed to minimize the energy consumption of the edge system in a device–edge/fog–cloud orchestrated network.

The problem of joint task-aware offloading and scheduling in MEC systems is studied in [22]. The proposed method formulates the problem as a mixed integer nonlinear program (MINLP) to schedule the offloaded co-tasks and minimize the average co-task completion time. In [23], the standard marine predators algorithm based on an energy-aware and delay-sensitive taskscheduling scheme is proposed to tackle the task scheduling in edge/fog computing and improve the QoSs required by IoT devices. In [24], an online algorithm named Dedas was proposed for the deadline-aware task dispatching and scheduling in edge computing to improve the number of completed tasks. These schemes were designed with a mathematical model and optimized through a heuristic algorithm and MINLP. Although the algorithms proposed in these schemes have a good performance, they are not adapted to the dynamic environment. In addition, because these schemes assume that the availability of computing resources is fixed, they are not adapted to the distributed edge environment which can improve the scalability of edge computing resources. The key differences between the relevant works and our work are shown in Table 1.

## 3. System Model and Problem Statement

In this section, we describe the proposed cluster edge computing system model. We also define the optimization problem of resource allocation for computation offloading services, considering the resource scalability and the resource optimization of edge computing. We first introduce the cluster-based edge computing system and then present the communication and the computation offloading models. Finally, we formulate the optimization problem with objective functions. For clarity, the major variables and notations used in our model are shown in Table 2.

### 3.1. Cluster Edge Computing System and Network Model

**Figure 1:**
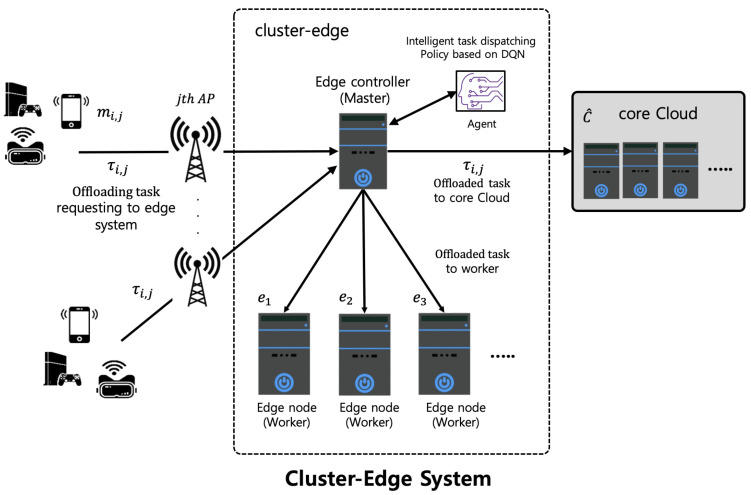
Cluster edge system-based network model.

We consider the network model with the cluster edge system as shown in Figure 1. We call the cluster edge system the cluster edge *e*. As shown in Figure 1, the cluster edge consists of an edge controller (a master) known as $e_{c}$ and *N* edge nodes (workers) known as $e_{n}$, which can execute an offloaded computation task. In the cluster edge, based on the procedures of a computation offloading, the mobile node (MN) requests task offloading to the cluster edge, and then the edge controller in the cluster edge determines the appropriate edge node (worker) with the application related to the offloaded task and sufficient computing resources. The offloaded task is forwarded to the selected edge node through a scheduler for task dispatching. After the task offloading is performed on the selected edge node, the edge controller collects and then responds to the task results for the MN, requesting task offloading. In this paper, we only focus on an edge node selection and scheduling for task dispatching among all edge nodes in the cluster edge.

As shown in Figure 1, we assume the edge-cloud network model in which the cluster edge is connected to the core Cloud collaborates in computation offloading. In the edge-cloud network model, the edge controller and *N* edge nodes are connected through a fiber switch, and the edge controller is connected to the core cloud through the backbone network. To support multiple services in the cluster edge, there may be multiple configured services. In the network model, we assume that there are the *i*th MN, $m_{i,j}$ connected to the *j*th Base Stations (BSs), and each BS serves multiple MNs within a radius. Multiple MNs offload tasks over wireless to the BSs, and then the BS sends the offloaded tasks to the cluster edge. After offloading the task at the cluster edge, it will be performed using a worker within the cluster edge. Then, the result of the offloaded task returns to MNs. Note that if the total computing resource of the cluster edge is insufficient to perform the offloaded task, the offloaded task is sent to the core cloud. We use an optical-fiber-based wired network between the edge controller and edge nodes in the cluster edge and between the cluster edge and the core cloud. That is why we assume that all the wired links have sufficient network resource bandwidth in the network model. Thus, in our work, we only consider the allocation of the wireless communication bandwidth denoted by $R_{i,j}$. For tasks based on AI applications, since the result data size of the offloaded task is much smaller compared with the data size of the offloaded task, we ignore the downlink bandwidth of wireless communication between the MNs and the cluster edge. To perform the offloaded task, the cluster edge stores the data of the offloaded task and then computes and analyzes the offloaded task. If an edge node in the cluster edge has sufficient computing resources, such as CPU cycles, it is assumed that each edge node can execute the offloaded task independently. In this paper, the computational resource needed to perform the task offloaded by the MN is characterized by the number of CPU cycles per second. Thus, to optimize the utilization of computing resources in the cluster edge and minimize the average task response time of all offloaded tasks, an efficient edge node (worker) selection and scheduling for task dispatching is required for the cluster edge.

### 3.2. Task Model

In this paper, we consider two types of task model: (1) single task, consisting of an application running with one micro-service, and (2) the bundle task, which consists of M independent multi-tasks running with multiple micro-services [25]. Thus, we assume that the bundle task can be partitioned into sub-tasks by the edge controller, and each sub-task partitioned from the bundle task can be independently performed through multi-edge nodes in the cluster edge.

The task offloaded from the MNs $m_{i,j}$ is expressed as the set $T_{i,j}$($ty_{i,j}$, $C_{i,j}$, $S_{i,j}$, $d_{i,j}$), where $ty_{i,j}$ is the type of the task described above, $C_{i,j}$ is the number of CPU cycles requested by the MN to perform the task, $S_{i,j}$ is the data size of the task, and $d_{i,j}$ is the task’s result deadline, such as the task response time requested by the MN. Here, if the $ty_{i,j}$ is a single task, $C_{i,j}$ and $S_{i,j}$ are defined as the value of one task and if the bundle task, CPU cycles space, $C_{i,j}$, can be given as $C_{i,j}=\left\{c\;|\;c=\left(c_{i,j}^{1},c_{i,j}^{2},\ldots ,c_{i,j}^{n}\right)\right\}$, and the sub-task data size space, $S_{i,j}$, can be given as $S_{i,j}=\left\{s\;|\;s=\left(s_{i,j}^{1},s_{i,j}^{2},\ldots ,s_{i,j}^{n}\right)\right\}$, where *n* is the number of sub-tasks in the bundle task. The task’s result deadline, $d_{i,j}$, is the most important consideration for the offloaded task of applications or services subject to strict time deadlines, e.g., virtual reality and real-time control systems. Additionally, the computational resources needed to perform $T_{i,j}$ are characterized by the number of CPU cycles $C_{i,j}$. Thus, in the case of a single task, $C_{i,j}$ is used as the required computing resource and, in the case of a bundle task, the total number of CPU cycles required by each sub-task in the bundle task is used as the required computing resource. Thus, to define the total number of CPU cycles requested by $T_{i,j}$ of the bundle task, assuming that the number of sub-tasks is *M*, the total number of CPU cycles required for $T_{i,j}$ of the bundle task is defined as $c_{i,j}^{M}$, given by

(1) $$\begin{array}{c} C_{i,j}\;=\;c_{i,j}^{M}\;=\sum_{n=1}^{M}c_{i,j}^{n}. \end{array}$$

The total data size of the bundle task is defined as $s_{i,j}^{M}$, given by

(2) $$\begin{array}{c} S_{i,j}\;=\;s_{i,j}^{M}\;=\sum_{n=1}^{M}s_{i,j}^{n}. \end{array}$$

As addressed above, in the cluster edge, the bundle tasks are partitioned into sub-tasks by the edge controller. Thus, the offloaded bundle tasks can be jointly performed through multiple edge nodes, which have sufficient computing resources in the cluster edge.

### 3.3. Computation Offloading Model

**Figure 2:**
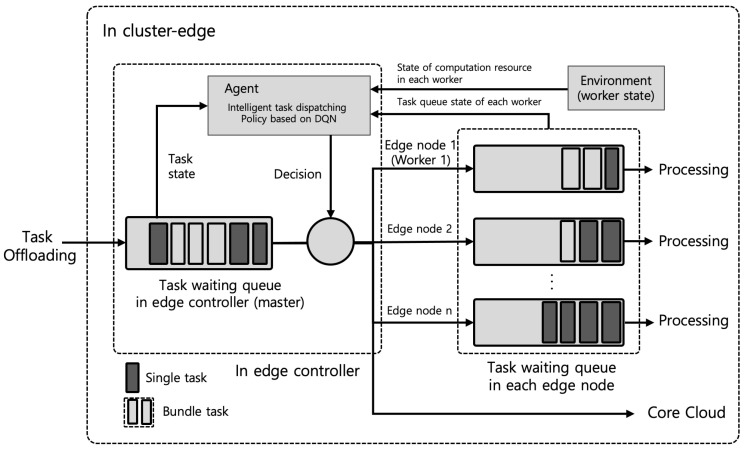
DRL-based intelligent task dispatching method (DDM) using DQN in the cluster edge.

In this paper, we assume that there is no local processing in the MN for computation offloading. Thus, in our scenario, there is only remote processing in both the cluster edge and core cloud. When the MN requests computation offloading with $T_{i,j}$ to the cluster edge, according to the computing resources required in $T_{i,j}$ and the current computing resource state of the cluster edge, the cluster edge performs the resource allocation procedure used for the required computation offloading. Here, we propose a DRL-based intelligence resource allocation model, called an intelligent task dispatch model, to optimally use the computational resources of *n* edge nodes in the cluster edge. Figure 2 shows the proposed intelligent task dispatching model based on the DRL for the cluster edge.

#### Intelligent Task Dispatching Model for Computation Offloading

As addressed above, the intelligent task dispatching model is used to select an optimal edge node (worker) from the all edge nodes in the cluster edge for $T_{i,j}$. In the proposed model, according to the type of the offloaded task, the selecting edge node’s decision is different. In the case of a single task, either one edge node or the core cloud is selected by the agent in Figure 2 for computing resource allocation. In the case of the bundle task, the agent decides on one of three decisions for computing resource allocation. One is that the agent selects one edge node, which is able to perform all sub-tasks of the bundle task with sufficient computing resources of more than $c_{i,j}^{M}$; another is that the agent selects multiple edge nodes, which are able to collaboratively perform each sub-task in the bundle task in a distributed manner; the other is that the agent selects the core cloud if the computing resources of the cluster edge are insufficient.

In this paper, the objective of the proposed model is to minimize the average task service delay for all tasks. Thus, to define the the average task service delay of all the offloaded tasks, we first describe the detailed task service delay, $D_{i,j}$, of each offloaded task, $T_{i,j}$, as the following. $D_{i,j}$ is classified into three parts: the task transmission delay, $D_{i,j}^{t}$; the task queuing delay, $D_{i,j}^{q}$; and the task computation processing delay, $D_{i,j}^{p}$. Therefore, the total task service delay is defined by $D_{i,j}=D_{i,j}^{t}+D_{i,j}^{q}+D_{i,j}^{p}$. To address this in detail, $D_{i,j}^{t}$ represents the transmission delay between the MN and the cluster edge connected through the BS, $D_{i,j}^{q}$ represents the queuing delay before task dispatching, and $D_{i,j}^{p}$ represents the processing delay for task processing. First, to define $D_{i,j}^{t}$, we consider the transmission time model used in [12]. In [12], assuming that the channel between an MN and the BS is a Rayleigh channel, the transmission bandwidth between the MN $m_{i,j}$ and *j*th BS can be defined as

(3) $$\begin{array}{c} R_{i,j}\;=\alpha_{i,j}^{B}\log_{2}\left(1+p_{i,j}^{C}h_{i,j}/N_{o}\right) \end{array}$$

where $p_{i,j}^{C}$ is the transmission power of $m_{i,j}$, $h_{i,j}$ is the channel gain, and $N_{o}$ is the variance in complex white Gaussian channel noise. For the analysis simplification, the transmission rate given by the above equation ignores the burst interference. Then, $D_{i,j}^{t}$ from $m_{i,j}$ to the cluster edge can be defined as

(4) $$\begin{array}{c} D_{i,j}^{t}\;=\frac{d_{i,j}}{R_{i,j}} \end{array}$$

where $d_{i,j}$ is defined in $T_{i,j}$ transferred from $m_{i,j}$.

When the computation offloading requested by $T_{i,j}$ arrives at the cluster edge, the cluster edge decides whether to offload the requested task to the core Cloud or to perform the requested task at the cluster edge based on the computing resource status of the cluster edge. Therefore, if the cluster edge does not have sufficient computational resources, it will offload the requested task to a core cloud, and if it has sufficient computational resources, it must decide on the appropriate edge node among *n* edge nodes to perform the requested task. Here, we do not consider the task transmission delay from the cluster edge to the core cloud because of its very small value as addressed above. To define $D_{i,j}^{q}$, we consider the queuing model in the framework of the proposed model, described in Figure 2. The framework is the new DRL-based intelligent task dispatching method (DDM) proposed in this paper. First, we define $D_{i,j}^{q}$ based on this model.

As shown in Figure 2, the DDM contains two kinds of queues at the cluster edge: task waiting queue for the task dispatching in the edge controller and task waiting queue for the task processing in the edge node. In more detail, $D_{i,j}^{q}$ can be defined by

(5) $$\begin{array}{c} D_{i,j}^{q}\;=D_{i,j}^{q^{c}}+D_{i,j}^{q_{n}^{e}}. \end{array}$$

where $D_{i,j}^{q^{c}}$ is the queuing delay in the task waiting queue of the edge controller, and $D_{i,j}^{q_{n}^{e}}$ is the queuing delay in the task waiting queue of *n*th edge node. $D_{i,j}^{q^{c}}$ is dependent on the decision processing time that occurs while selecting the edge node for task dispatching by the agent. Accordingly, $D_{i,j}^{q^{c}}$, after arriving at the task waiting queue of the edge controller, can be calculated and defined by

(6) $$\begin{array}{c} D_{i,j}^{q^{c}}\;=avg\left(DPT\right)\left(N\right) \end{array}$$

where DPT is the decision processing time needed to select an edge node for task dispatching, and *N* is the total number of both all single tasks and all sub-tasks of the bundle task, which is waiting in the waiting queue when $T_{i,j}$ arrives at the waiting queue of the edge controller. We used the average decision processing time, avg(DPT), as the decision processing time. $D_{i,j}^{q_{n}^{e}}$ can be estimated after the agent’s decision to select the edge node for $T_{i,j}$. Thus, after the agent’s decision, $T_{i,j}$ forwards to the task waiting queue of the selected edge node. $D_{i,j}^{q_{n}^{e}}$ is dependent on the number of tasks waiting in the waiting queue and total computation processing time needed to process all tasks in the waiting queue, when $T_{i,j}$ arrives at the waiting queue of *n*th edge node. Thus, $D_{i,j}^{q_{n}^{e}}$ can be defined by

(7) $$\begin{array}{c} D_{i,j}^{q_{n}^{e}}\;=\sum_{m=1}^{N}P_{m} \end{array}$$

where *N* is the total number of tasks, including all single tasks and all sub-tasks of the bundle task, and $P_{m}$ is the computation processing time of *m*th task in the task waiting queue of *n*th edge node. To estimate $P_{m}$ at *n*th edge node, first, in cases where there is a single task in the waiting queue, its computation processing delay can be estimated by

(8) $$\begin{array}{c} P_{m}\;=\frac{c_{i,j,m}}{\alpha_{i,j}^{n}f^{n}}. \end{array}$$

where $c_{i,j}$ is defined in $T_{i,j}$, transferred from $m_{i,j}$, $\alpha_{i,j}^{n}$ is the ratio of the computing resource provided by *n*th edge node to perform $T_{i,j}$, and $f^{n}$ is the total computing resource of *n*th edge node, which is denoted as the CPU cycles. In the case of the sub-task of the bundle task in the waiting queue, its computation processing delay can be estimated by

(9) $$\begin{array}{c} P_{m}\;=\frac{c_{i,j,m}^{k}}{\alpha_{i,j}^{n}f^{n}}. \end{array}$$

where $c_{i,j}^{k}$ is defined as the requested computing resource of the *k*th sub-task in $T_{i,j}$, set as a bundle task. Moreover, assuming that there are total *N* tasks, which consist of a total number of single tasks, *S*, and a total number of sub-tasks, *R*, in the waiting queue of the *n*th edge node, the queuing delay at the *n*th edge node, as addressed in Equation (7), $D_{i,j}^{q_{n}^{e}}$, can be estimated by

(10) $$\begin{array}{c} D_{i,j}^{q_{n}^{e}}\;=\sum_{m=1}^{S}\frac{c_{i,j,m}}{\alpha_{i,j}^{n}f^{n}}\;+\;\sum_{m=1}^{R}\frac{c_{i,j,m}^{k}}{\alpha_{i,j}^{n}f^{n}}. \end{array}$$

Finally, to estimate the processing delay for task processing, $D_{i,j}^{p}$ for $T_{i,j}$ can use Equation (8) for a single task and Equation (9) for the sub-task of a bundle task.

Thus, in the case of a single task, the expected task service delay, $D_{i,j}$, for $T_{i,j}$ can be represented by

(11) $$\begin{array}{c} \begin{array}{cc} D_{i,j} & \;=D_{i,j}^{t}+D_{i,j}^{q}+D_{i,j}^{p}\\ \; & \;=\frac{d_{i,j}}{R_{i,j}}\;+\;\left(avg\left(TD\right)\left(N\right)\;+\;\sum_{m=1}^{S}\frac{c_{i,j,m}}{\alpha_{i,j}^{n}f^{n}}\;+\;\sum_{m=1}^{R}\frac{c_{i,j,m}^{k}}{\alpha_{i,j}^{n}f^{n}}\right)\;+\;\frac{c_{i,j}}{\alpha_{i,j}^{n}f^{n}}. \end{array} \end{array}$$

and in the case of a bundle task, the total *k*th sub-task service delay, $D_{i,j,k}$ can be represented by

(12) $$\begin{array}{c} \begin{array}{cc} D_{i,j,k} & \;=D_{i,j,k}^{t}+D_{i,j,k}^{q}+D_{i,j,k}^{p}\\ \; & \;=\frac{d_{i,j}}{R_{i,j}}\;+\;\left(avg\left(TD\right)\left(N\right)\;+\;\sum_{m=1}^{S}\frac{c_{i,j,m}}{\alpha_{i,j}^{n}f^{n}}\;+\;\sum_{m=1}^{R}\frac{c_{i,j,m}^{k}}{\alpha_{i,j}^{n}f^{n}}\right)\;+\;\frac{c_{i,j}^{k}}{\alpha_{i,j}^{n}f^{n}}. \end{array} \end{array}$$

Thus, we assume that there are *K* sub-tasks in the bundle task and a bundle task is performed in a distributed manner in the cluster edge. The expected bundle task service delay can be represented by

(13) $$\begin{array}{c} D_{i,j}^{b}\;=\frac{\sum_{k=1}^{K}D_{i,j,k}}{K} \end{array}$$

### 3.4. Optimization Problem Formulation

We formulate the optimization problem to minimize the average task service delay of all tasks offloaded from MNs. We first formulate the problem of minimizing the average task service delay ${\tilde{D}}_{T_{i,j}}$, as shown in Equation (14). The task service delay is the time taken to complete the task initiated at time tau within the range of *j*th BS under the cluster edge. We assume that the MNs do not experience the channel interference for other BS in a single BS, and the wireless link bandwidth is equal to R. The wireless link bandwidth is limited by these constraints.

(14) $$\begin{array}{c} \begin{array}{cc} {\tilde{D}}_{i,j}\; & =min\frac{1}{NM}\sum_{i=1}^{N}\sum_{j=1}^{M}\left(D_{i,j}+D_{i,j}^{b}\right)\\ \; & =min\frac{1}{NM}\sum_{i=1}^{N}\sum_{j=1}^{M}(\frac{d_{i,j}}{R_{i,j}}\;+\;avg\left(TD\right)\left(N\right)\;+\;\sum_{m=1}^{S}\frac{c_{i,j,m}}{\alpha_{i,j}^{n}f^{n}}\;+\;\sum_{m=1}^{R}\frac{c_{i,j,m}^{k}}{\alpha_{i,j}^{n}f^{n}}\;+\;\frac{c_{i,j}}{\alpha_{i,j}^{n}f^{n}}\\ \; & \;\;+\frac{\sum_{k=1}^{K}\left(\frac{d_{i,j}}{R_{i,j}}\;+\;avg\left(TD\right)\left(N\right)\;+\;\sum_{m=1}^{S}\frac{c_{i,j,m}}{\alpha_{i,j}^{n}f^{n}}\;+\;\sum_{m=1}^{R}\frac{c_{i,j,m}^{k}}{\alpha_{i,j}^{n}f^{n}}\;+\;\frac{c_{i,j}^{k}}{\alpha_{i,j}^{n}f^{n}}\right)}{K}) \end{array} \end{array}$$

under the constraints

$$\begin{array}{c} \sum_{i=1}^{N}R_{i}\;\leq \;R,\;R\;\;\geq \;0,\;\forall_{i} \end{array}$$

## 4. Drl-Based Task Dispatching Method in the Cluster Edge

In this section, for simplicity, we consider an offloading task with edge applications deployed on the cluster edge and focus on task dispatching for the resource allocation to process the tasks generated by the application in MN. Thus, we propose a DRL-based task dispatching method. The proposed DRL-based task dispatching applies the deep Q-network (DQN) for policy training, which exploits past experience based on edge node selection by estimating the current state of the environment based on the load state of the edge node, to select the edge node for offloading tasks in the cluster edge. The objective of our work is to increase task offloading and to reduce the service latency of the processing of offloaded tasks, which will allow us to improve the utilization of limited resources in the cluster edge. To fulfill these objectives, we formulate the above problem as a Markov Decision Process (MDP) as τ = 0, 1, ……, *∞*.

### 4.1. Markov Decision Process

In general, the MDP model contains several elements: agent, state, action, policy, and reword. In our intelligent task dispatching model, the agent has the role of interacting with an environment, which is known as a state. The state is defined as the status of the computing resource in the cluster edge and status information of the offloaded task. The action refers to edge node selection to allocate the computing resource of the offloaded tasks by the agent playing the role of resource allocation, based only on the current state with an optimization function. The policy is the function of the pair of state and action (s,a). The reward is defined as the response of the pre-formed action and is received from the environment. The detailed MDP model is explained as follows. The agent has the role of task dispatching, choosing the edge node of the offloaded task based on the current state of the environment. Thus, the goal of the agent is to make the optimal decision in each round to minimize the overall average task service delay for tasks in τ. The state space $s_{\tau }\in \;S$ is defined as the state of edge nodes in the cluster edge and the state of task requested in the edge network, which can be given as $S\;=\{s\;|\;s=\left(D^{q^{c}},D^{q_{1}^{e}},D^{q_{2}^{e}},\ldots ,D^{q_{n}^{e}}\right)\}$, where $D^{q^{c}}$ represents the queuing delay in the edge controller and $D^{q_{1}^{e}}$∼$D^{q_{n}^{e}}$ represent the queuing delay in each edge node, where *n* is the number of edge nodes needed to perform the computation offloading service in the cluster edge. In our model, the agent used for task dispatching will take action by observing the current state of the environment. Thus, the action of agent $a_{\tau }$ is to select the edge node for the task in this round, which indicates that the computing resource of the selected edge node will be assigned to the current task. Let $A=\{a\;|\;a=\left(e_{1},e_{2},\ldots ,e_{n}\right)\}$ be an action space, where $e_{n}$ is *n*th edge node and *n* is the total number of edge nodes. The state is transferred from $s_{\tau }$ to $s_{\tau +1}$ through action $a_{\tau }$ with the probability $P(s_{\tau +1}\;|\;s_{\tau },a_{\tau })$. The action policy $\pi (s_{\tau }):S\to A$ defines the mapping relationship from the state to the action. The policy is updated by training the agent. The task dispatching policy indicates a set of actions $a_{\tau }=\pi \left(s_{\tau }\right)$, which maps the state to an action at time τ. After observing the state of the queuing delay in the edge controller and in each edge node at time τ, the agent will take action according to the task dispatching policy based on the current state and then receive the reward from the environment at time τ+1. The received reward will be used to update the task dispatching policy to make a decision regarding the optimal action for the next action. In RL, since the agent’s goal is to maximize the objective, which is the sum of rewards achieved by taking good actions, it learns to choose the optimal action by interacting with the environment. The detailed design of the reward function used in our model is explained as follows. In our work, the optimization objective problem or function is to minimize the average task service delay of all offloaded tasks so that each action is optimal. At time τ, the agent will observe the current state in all task waiting queues in the edge controller and all edge nodes and will select the edge node optimally using the action a(τ) based on the policy iteration. It will then evaluate the performance of the action using the following reward function:

(15) $$R\left(s\left(\tau \right),a\left(\tau \right)\right)\;=\begin{array}{cc} \exp({\tilde{D}}_{i,j}-D_{i,j}) & \mathrm{if}\;{\tilde{D}}_{i,j}>D_{i,j}\\ -\exp(D_{i,j}-{\tilde{D}}_{i,j}) & \mathrm{if}\;D_{i,j}>{\tilde{D}}_{i,j} \end{array}$$

where ${\tilde{D}}_{i,j}$ is the average service delay of all tasks, and $D_{i,j}$ is the estimated service delay of the current task. In our model, the objective function is to minimize the average task service delay. Thus, in the reward function design, we use average task service delay as the baseline and make the current task service delay as small as possible through the exponential function. If the agent uses a good action for the offloaded tasks, the average service delay will be small. A good policy allows for most offloading tasks to be performed with a sufficient service delay without exceeding the deadline. Finally, we define the optimization objective or the reward. At time τ, the agent estimates the performance of the current action using the reward returned by the reward function, R{s(τ),a(τ)}. Thus, for DRL-based learning, to maximize the expected cumulative discounted reward, the optimization objective is defined as

(16) $$\begin{array}{c} \max_{a_{\tau }}E[\sum_{\tau =0}^{\sigma }\gamma^{2}R\left(s\left(\tau \right),a\left(\tau \right)\right], \end{array}$$

where γ∈(0,1] is the factor discounting future rewards. In addition, the optimization objective minimizes the average completion time of all offloaded tasks and the utility of computational resources in the long term.

### 4.2. A Drl-Based Task Dispatching Method Using DQN

**Figure 3:**
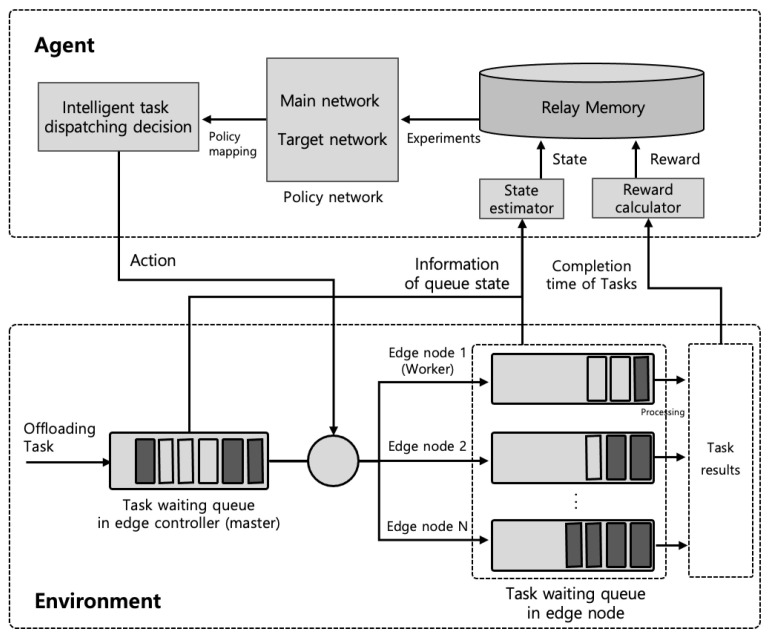
Illustration of the proposed DQN for task dispatching policy.

In our model, we used the deep Q-network (DQN) algorithm for the learning process. The DQN algorithm is an off-policy algorithm, which does not have to discard experiences once they have been used, and also a value-based temporal difference (TD) algorithm, which can approximate an action–value function called a Q-function. Thus, the agent decides on the optimal action through the learned optimal Q-function instead of the Q-function based on the current policy, compared to traditional Q-learning such as SARSA. DQN is applied to environments with discrete action spaces. However, the model in this paper assumes that the state space and the action space of environments are continuous and large. Thus, to solve the optimal problem assumed in this paper, we applied the DQN model and the convectional neural network (CNN) used in [11]. The learning process for DQN is shown in Figure 3.

As shown in Figure 3, the proposed learning process based on DQN applies an experience replay memory (ERM), which is able to store experience data and then use this for learning. Here, the experience replay memory is used to solve the problem related to the correlation between the experience data. The experience replay memory stores the most recent experience, e=(s(τ),a(τ),R(s(τ),a(τ)),s(τ+1)), which an agent gathers by interacting with the environment. If it is full, the oldest experience is discarded. The agent will randomly sample a mini-batch of data from the experience replay memory every *b* time the agent trains; then, it will update the network’s parameter, θ, of the Q-function network with each min-batch and stochastic gradient descent (SGD), as defined by

(17) $$\begin{array}{c} \theta_{i+1}=\theta_{i}-\sigma {▽}_{\theta }Loss\left(\theta \right) \end{array}$$

where σ is the learning rate.

Differing from Q-learning, DQN uses two neural networks, which comprise the main network Q(s,a;θ) and the target network $\tilde{Q}$. These networks have the same network structure but different network parameters called the Q-value. Here, one is the current Q-value θ generated in the main network, called the prediction network, and the other is the target Q-value $\tilde{\theta }$ generated in the target network. As addressed above, DQN is a value-based TD algorithm and involves an action–value function (called Q-function). Therefore, for a particular policy π, the action–value function $Q^{\pi }\left(s,a\right)$ measures the value of state–action pairs (s,a) and is defined as

(18) $$\begin{array}{c} Q^{\pi }\left(s,a\right)=E[\sum_{\tau =0}^{\sigma }\gamma^{2}R\left(s\left(\tau \right),a\left(\tau \right)\right], \end{array}$$

which is the same as (17), defined to estimate the performance of the current action by the returned reward value. To obtain the optimal policy $\pi^{*}$, which is defined to be better than or equal to a current policy π, the optimal Q-function is defined as taking action *a* in state *s*, as follows

(19) $$\begin{array}{c} Q^{*}\left(s,a\right)=max_{\pi }Q^{\pi }\left(s,a\right)=Q^{\pi^{*}}\left(s,a\right), \end{array}$$

in which, if the estimation of $Q^{\pi }\left(s,a\right)$ is correct, the action that maximizes $Q^{\pi }\left(\overset{´}{s},\overset{´}{a}\right)$ will be optimal. Thus, the optimal policy $\pi^{*}$ is given by

(20) $$\begin{array}{c} \pi^{*}\left(a|s\right)=argmax_{a\in \;A(s)}Q^{*}\left(s,a\right). \end{array}$$

Thus, the optimal Q-function can be rewritten with the Bellman equation as follows

(21) $$\begin{array}{c} Q^{*}\left(s,a\right)=E_{\pi }\left[r\left(s,a\right)+\gamma max_{\overset{´}{a}}Q^{*}\left(\overset{´}{s},\overset{´}{a}\right)\right], \end{array}$$

where γ∈(0,1] is a factor discounting future rewards. Based on (22), the optimal Q-function can be estimated with the loss function, defined as follows

(22) $$\begin{array}{c} Q\left(s,a\right)\leftarrow Q\left(s,a\right)+\alpha [r\left(s,a\right)+\gamma max_{\overset{´}{a}}Q\left(\overset{´}{s},\overset{´}{a}\right)-Q\left(s,a\right)], \end{array}$$

where Q(s,a) is the learned Q-value, $r\left(s,a\right)+\gamma max_{\overset{´}{a}}Q\left(\overset{´}{s},\overset{´}{a}\right)$ is the estimated Q-value, and α is the learning rate. Differing from the Q-function, DQN uses a neural network Q(s,a;θ), called the main network, as the approximation function to estimate Q-function. Thus, the loss function for DQN is defined by

(23) $$\begin{array}{c} L\left(\theta \right)=\frac{1}{N}\sum_{e\in N}\left[{\left(\tilde{Q}-Q\left(s,a;\theta \right)\right)}^{2}\right], \end{array}$$

where *e* is experience, *N* is a mini-batch of experiences, and $\tilde{Q}$ is given by

(24) $$\begin{array}{c} \tilde{Q}=E_{\pi }\left[r\left(s,a\right)+\gamma max_{\overset{´}{a}}Q\left(\overset{´}{s},\overset{´}{a};\tilde{\theta }\right)\right], \end{array}$$

where $\tilde{Q}$ updates by copying from θ every τ iteration time.

The proposed intelligent task dispatching algorithm using DQN is described in Algorithm 1. The agent makes a random decision using the random algorithm at the learning start time. However, through the iterative processes and learning and policy updates, the proposed algorithm finds the optimal policy. Thus, at the end of the iterations for learning and updating, the agent will take the learned optimal policy and can make the optimal decision.
**Algorithm 1** Intelligent task dispatching algorithm in the cluster edge     **Input:** the number of edge node, computing ability of edge nodes, radio bandwidth               resource, parameters for the task setting     **Output:** edge node selection a(τ)1:Initiate learning rate σ;2:Initiate τ;3:Initiate the number of mini-batches *B*;4:Initiate batch size *N*;5:Initiate experience replay memory with max size *K*;6:Initiate main network *Q* with random parameter θ;7:Initiate target network $\tilde{Q}$ with parameter $\tilde{\theta }$ = θ;8:**for** episode e=1…MaxSteps **do**9:    **for** τ = 1:*T* **do**10:        Get the current state $s_{\tau }$ from the environment;11:        Take action12:        a(τ) = $\left\{\begin{array}{c} \mathrm{random}\;\mathrm{action}\;\mathrm{from}\;A(s),\;\mathrm{prob}.\;\varepsilon \\ argmax_{a\in A(s)},Q\left(s,a;\theta \right),1-\varepsilon ; \end{array}\right.$13:        Perform a(τ), receive r(τ) and perform state transition s(τ)→s(τ+1);14:        Gather and store experiences e=(s(τ),a(τ),r(τ),s(τ+1)) using the current15:         policy into ERM;16:        **for** b=1…B **do**17:           Randomly sample a mini-batch *b* of experiences from ERM18:           **for** i=1…N **do**19:               # Calculate target Q-values for each example20:               $y_{i}=r_{i}+$$\delta_{\overset{´}{s_{i}}}\gamma max_{{\overset{´}{a}}_{i}}Q^{\pi }\left(\overset{´}{s_{i}},\overset{´}{a_{i}};\tilde{\theta }\right)$ where $\delta_{\overset{´}{s_{i}}}=0$ if $\overset{´}{s_{i}}$ is terminal,21:                  -> 1 otherwise;22:           **end for**23:           Calculate the loss L(θ) by (23);24:           Update the network’s parameters θ by (17);25:           Set $\tilde{Q}=Q$;26:        **end for**27:    **end for**28:    Decay τ29:**end for**

## 5. Performance Evaluation and Comparison

In this section, we evaluate the performance of the proposed DQN-based task dispatching method and compare this with a static scenario with static nodes and a mobile scenario with mobile nodes. We consider a scenario consisting of one cluster edge and three BS, with a circle area of 1000 m${}^{2}$. For this, we develop a mathematical model-based simulator based on Pytorch-1.9. The simulations for a performance evaluation are to validate the mathematical formulation and the DQN based algorithm for task dispatching. In the simulation, we assume that all applications performing all offloaded tasks are deployed on all edge nodes in the cluster edge. In addition, all static and mobile nodes are scattered uniformly in the BS coverage and the speed of mobile nodes is randomly chosen between 20 m${}^{2}$ and 120 m${}^{2}$. The transmission power is selected in a range from 32 mW to 197 mW through channel gain $h_{i,j}$. The channel number at BS is 20, and the bandwidth of a channel is 2 MHz. For the computation task, we consider the object detection as a target application, which relies on the devices used to collect images and offload them to the cluster edge. These offloaded tasks are service-latency-sensitive and computation intensive. Thus, we set the data size of the task as high load-input data to heighten the computing requirements. Additionally, the required CPU cycles for each task are randomly assigned from $2.6\times 10^{9}$ to $5.2\times 10^{9}$. To set the task, the number of sub-tasks in the bundle task follows a discrete uniform distribution, with a range from 2 to 4. The arrival rate of all tasks follows a Poisson distribution with a mean rate of λ = 30. This means that there is an average of 30 tasks arriving at the cluster edge per time slot. The main simulation parameters related to the environment are shown in Table 3, and the hyperparameters for DQN learning are shown in Table 4.

To estimate the performance of the proposed model, we compare the proposed model with three existing dispatching methods in terms of average task service delay and average task completion rate:

- Random Method (RM): Dispatch the offloaded task to the randomly elected edge node;
- Least Load Method (LLM): Dispatch the offloaded task to the edge node with minimal waiting queue time;
- Round-Robin Method (RRM): Dispatch the offloaded task in the sequence of edge node.

**Figure 4:**
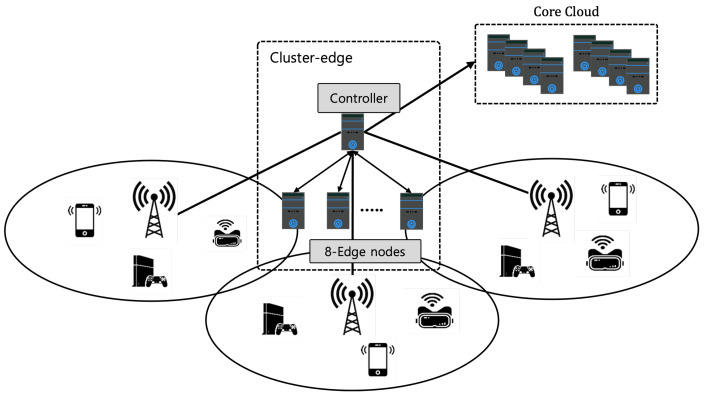
Network model of simulation scenario with static nodes.

We evaluate the performance of the proposed DDM model in the network model shown in Figure 4, which consists of 100-static nodes, one cluster edge computing system, three BS, and a cloud.

**Figure 5:**
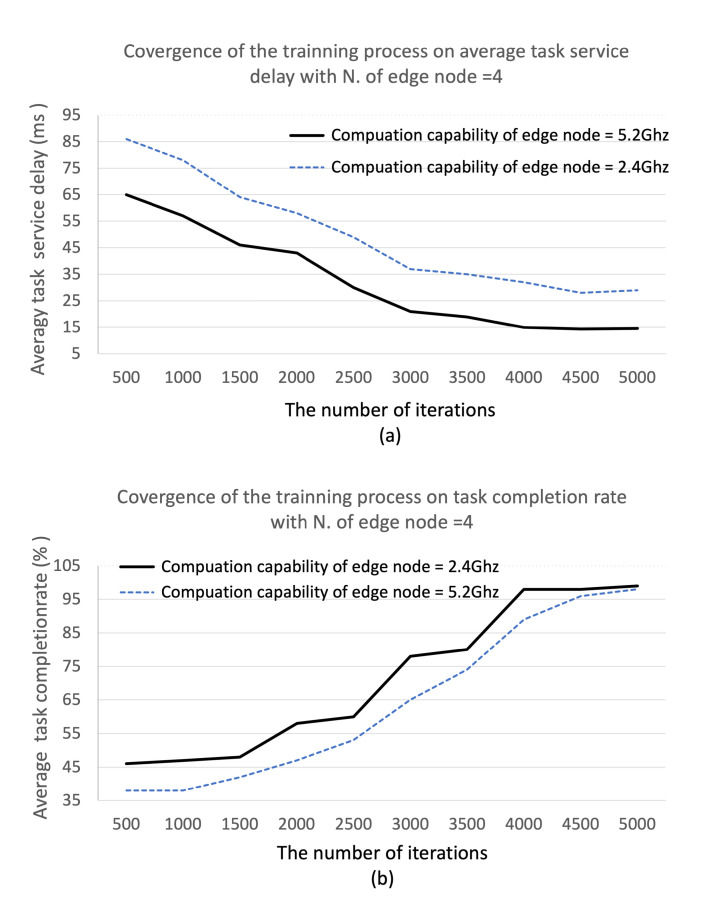
The convergence performance of the proposed DDM model on: (**a**) average task service delay; (**b**) task completion rate with the number of edge node = 4.

**Figure 6:**
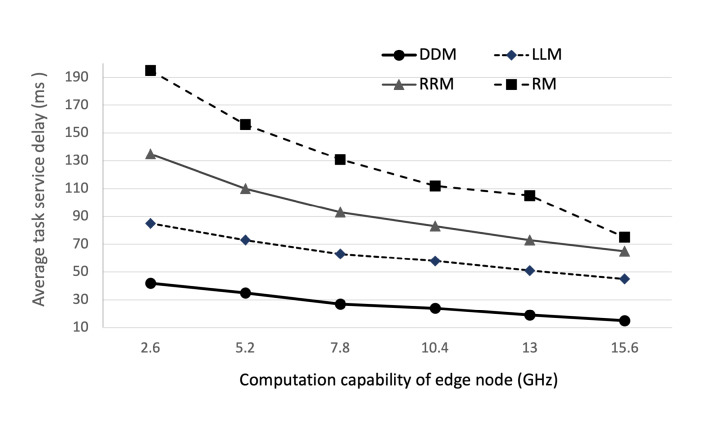
Average task service delay according to the computation capacity of edge nodes.

**Figure 7:**
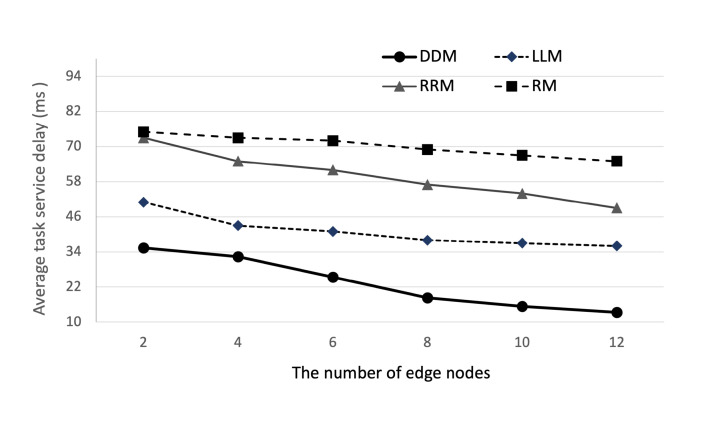
Average task service delay according to the number of edge nodes.

We first examine the convergence performance of the proposed DDM model with the number of iterations. As shown in Figure 5a, the average task service delay decreases in the 4000 iterations and enters a stable status and also, as shown in Figure 5b, in the 4000 iterations, average task completion rate converges to 97%∼99% as the training proceeds, showing that the algorithm in the proposed DDM model will reach convergence. Then, we evaluate the impact of the computation ability of edge nodes and the number of edge nodes in the cluster edge on the average task service delay. As shown in Figure 6 and Figure 7, we set the computation capacity from 2.6 GHz to 15.6 GHz and the number of edge nodes from 2 to 12. The proposed DDM model compares the performance with the Random Method (RM), the Least Load Method (LLM), and the Round-Robin Method (RRM). As expected, the simulation results show that average task service delay decreases according to the increase in the computational ability and the number of edge nodes. Furthermore, regarding computational ability, the proposed DDM model improves the performance compared to the total average value of average task service delay by 55%, 70%, and 78% compared with RM, LLM, and RRM, respectively. Regarding the number of average edge nodes, the proposed DDM model improves the performance compared to the total average value of average task service delay by 43%, 61%, and 66% compared with RM, LLM, and RRM, respectively. Specifically, we see that the average task service delay of the proposed DDM model decreases from 35 ms to 13 ms when the number of edge nodes increases from 2 to 12. These results are because the proposed DDM model performs the load-balancing of the offloaded tasks well. We also observe that the offloading task ratio increases as the computational utility of edge nodes in the cluster edge increases. This shows that the DDM model can offload more tasks to the cluster edge.

**Figure 8:**
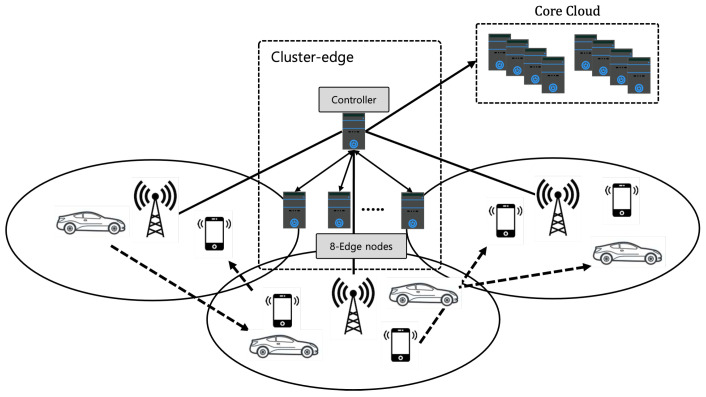
Network model of simulation scenario with mobile nodes.

Next, we evaluate the performance of the cluster edge in mobile environments, as shown in Figure 8, which consists of $N_{M}$ mobile nodes, one cluster edge computing system with eight edge nodes, three BS, and a cloud. We assume that the network supports the data handover between two BSs and only considers the change in channel quality during the mobile nodes’ handover. In this simulation, the number of edge nodes is eight and the computational ability is 10.4 GHz.

**Figure 9:**
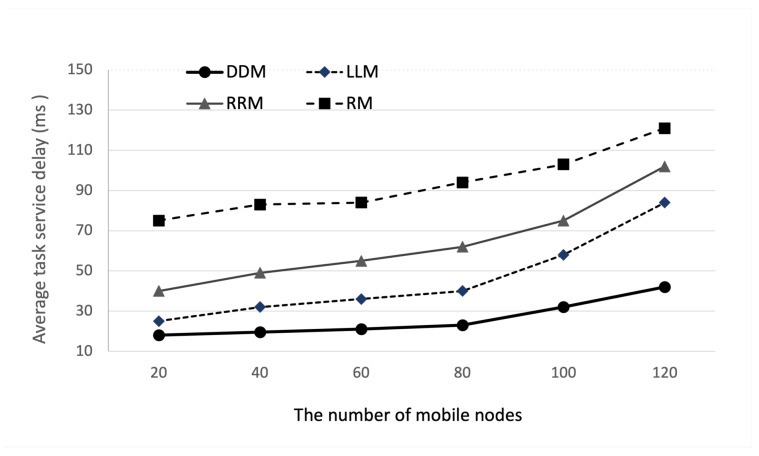
Average task service delay according to the number of mobile nodes.

**Figure 10:**
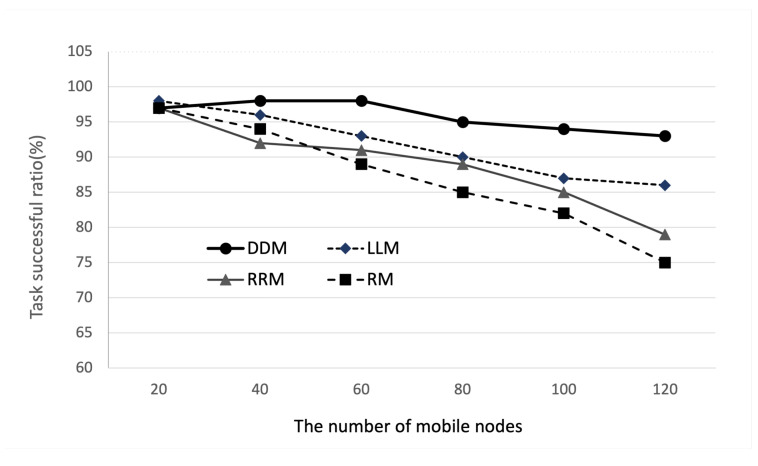
Task successful ratio according to the number of mobile nodes.

Figure 9 and Figure 10 show the impact of the number of mobile nodes on the average service latency and task successful ratio. As shown in the simulation results in Figure 9, the average task service delay of the DDM model increases from 18 ms to 42 ms when the number of mobile devices increases from 20 to 120. Additionally, compared with RM, LLM, and RRM, we can observe that the proposed DDM model improves the performance compared to the total average value of average task service delay by 43%, 59%, and 72%, respectively. This result is because the proposed DDM model distributes the offloaded task well in a dynamic environment. In addition, as shown in the simulation results in Figure 10, we can observe that the task successful ratio according to the number of mobile nodes decreases from 97% to 93%. Compared with RM, LLM, and RRM, the proposed DDM model improves the performance on the total average value by 4%, 6%, and 7%, respectively. This is mean that the proposed DDM model has a better performance than existing traditional methods and is an intelligent task dispatching policy, which can adjust well in dynamic edge service environments. As part of future works, we plan to extend our DDM model and then evaluate the extended model with a cluster emulator as the Fogify [26] in a real environment.

## 6. Conclusions

In this paper, we investigate the task dispatching policy for resource optimization in the cluster edge system. First, we have formulated the optimization problem related to the resource allocation policy for the cluster edge system. The formulated problem is based on the Markov decision process (MDP), which is solved by our proposed deep Q-network (DQN) optimization algorithm. In addition, we propose a DRL-based intelligent task dispatching method (DDM) for task load balancing in the cluster edge. The proposed DDM model uses a DQN algorithm as the DRL technology and the efficient resource allocation policy optimized for the resource management according to the state of edge nodes on the cluster edge. With the simulation, we show that the proposed DDM model can achieve a better performance than the existing methods in terms of the offloaded task service delay and an offloaded task completion rate. In addition, the simulation results show the optimal performance on the utility of computing resource in the cluster edge system in static and dynamic environments. This proves that the proposed DDM model can obtain a better convergence performance regarding average task service delay and average task completion rate and achieve a smaller sum of average completion time for tasks as our optimization objective. As part of future works, we plan to extend our DDM model with resource consideration which includes CPU, memory, cache, and network performance. In addition, we will evaluate the extended model with cluster emulator as the Fogify in real environment.

## Figures and Tables

**Table 1:** Comparison of relevant works.

Work	Objective	Algorithm	Environments
Our work	Average task service delay and average task completion rate for cluster edge	DQN	Static, Dynamic
[9]	Average task service delay for collaborative edge	SARSA	Static, Dynamic
[10]	Task service delay for distributed edge	DDPG	Dynamic
[11]	Resource utilization for an edge	DQN	Static
[12]	Average task service delay for collaborative edge	MCTS	Static, Dynamic
[13]	Task satisfaction degree for an edge	Q-network	Static, Dynamic
[18]	Service Migration Optimization for collaborative edge	Multi-Agent DRL	Static, Dynamic
[20]	Average task service delay for an edge	DRL	Dynamic
[21]	Energy consumption for an edge	DRL	Dynamic

**Table 2:** Variables and notations used in our model.

Notation	Definition
$m_{i,j}$	The *i*th MN at the *i*th BS
$e_{c}$	The edge controller in the cluster edge
$e_{n}$	*n*th edge node in the cluster edge
$\hat{C}$	Collaborative core Cloud
$T_{i,j}$	Task of $m_{i,j}$ offloaded to the cluster edge
$ty_{i,j}$	The type of the task $T_{i,j}$
$c_{i,j}$	The number of CPU cycles requested in the single task
$c_{i,j}^{k}$	The number of CPU cycles requested *k*th sub-task in the bundle task
$s_{i,j}$	The data size of the single task
$s_{i,j}^{k}$	The data size of *k*th sub-task in the bundle task
$d_{i,j}$	The task’s result deadline in the task required by $m_{i,j}$
$R_{i,j}$	The wireless link bandwidth between *i*th MN and *i*th BS
$p_{i,j}^{C}$	The transmit power of $m_{i,j}$
$h_{i,j}$	The channel gain of MN and *j*th BS
$D_{i,j}$	Task service delay of $T_{i,j}$
$D_{i,j,k}$	*k*th sub-task service delay of $T_{i,j}$ set as the bundle task
$D_{i,j}^{b}$	Bundle task service delay of $T_{i,j}$
$D_{i,j}^{t}$	The task transmission delay
$D_{i,j}^{q}$	The task queuing delay
$D_{i,j}^{p}$	The task computation processing delay
$D_{i,j}^{q^{c}}$	The queuing delay in the task waiting queue of edge controller
$D_{i,j}^{q_{n}^{e}}$	The queuing delay in the task waiting queue of *n*th edge node
$P_{m}$	The computation processing time of *m*th task
$f^{n}$	The total computing resource of *n*th edge node
$D^{q^{c}}$	The queuing delay in edge controller
$D^{q_{1}^{e}}$∼$D^{q_{n}^{e}}$	The queuing delay in *n*th edge node
${\tilde{D}}_{T_{i,j}}$	The average task service delay

**Table 3:** Main simulation parameters for the environment.

Parameters	Description	Value
$N_{S}$	The number of static nodes for non-mobility scenario	50
$N_{M}$	The number of mobile nodes for mobility scenario	20∼120
*M*	The number of sub-tasks in bundle task	2∼4
*N*	The number of edge nodes in cluster edge	5∼10
$s_{i,j}$	The data size of the task	200kB∼5MB
$c_{i,j}$	The total number of CPU cycles requested to serve task	$2.6\times 10^{9}∼5.2\times 10^{9}$
$d_{i,j}$	The tolerant service delay of offloading task required by $m_{i,j}$	5ms∼50ms

**Table 4:** The hyperparameters for DQN learning.

Parameters	Description	Value
episode,e	The number of iterations	5000
σ	Learning rate	0.005
*K*	The size of experience replay memory	10.000
*B*	The number of mini-batches	8
*N*	The size of mini-batches	32
γ	Factor discounting future rewards	0.9
τ	Step parameters	1500

## Data Availability

Not applicable.

## Abbreviations

The following abbreviations are used in this manuscript:

AI	Artificial Intelligence
AR	Augmented Reality
BS	Base Station
DDM	DRL based intelligent task Dispatching Method
DNN	Deep Neural Network
DPT	Decision Processing Time
DQN	Deep Q-Network
DRL	Deep Reinforcement Learning
ERM	Experience Replay Memory
LLM	Least Load Method
MDP	Markov Decision Process
MEC	Mobile Edge Computing
MN	Mobile Node
RM	Random Method
RRM	Round-Robin Method
QoS	Quality of Service
VR	Virtual Reality
